# Correlates of Non-suicidal Self-Injury and Suicide Attempts in Bulimic Spectrum Disorders

**DOI:** 10.3389/fpsyg.2016.01244

**Published:** 2016-08-22

**Authors:** Alexandra Gómez-Expósito, Ines Wolz, Ana B. Fagundo, Roser Granero, Trevor Steward, Susana Jiménez-Murcia, Zaida Agüera, Fernando Fernández-Aranda

**Affiliations:** ^1^Department of Psychiatry, University Hospital of Bellvitge-IDIBELLBarcelona, Spain; ^2^Ciber Fisiopatologia Obesidad y Nutrición (CIBEROBN), Instituto Salud Carlos IIIBarcelona, Spain; ^3^Departament de Psicobiologia i Metodologia. Universitat Autònoma de BarcelonaBarcelona, Spain; ^4^Department of Clinical Sciences, School of Medicine, University of BarcelonaBarcelona, Spain

**Keywords:** bulimic spectrum disorder (BSD), emotion regulation, personality, impulsivity, non-suicidal self-injury (NSSI), suicide attempts

## Abstract

**Objective:** The aim of this study was to examine the implication of personality, impulsivity, and emotion regulation difficulties in patients with a bulimic-spectrum disorder (BSD) and suicide attempts (SA), BSD patients with non-suicidal self-injury (NSSI), and BSD patients without these behaviors.

**Method:** One hundred and twenty-two female adult BSD patients were assessed using self-report questionnaires. Patients were clustered *post-hoc* into three groups depending on whether they presented BSD without NSSI or SA (BSD), BSD with lifetime NSSI (BSD + NSSI) or BSD with lifetime SA (BSD + SA).

**Results:** The BSD + NSSI and BSD + SA groups presented more emotion regulation difficulties, more eating and general psychopathology, and increased reward dependence in comparison with the BSD group. In addition, BSD + SA patients specifically showed problems with impulse control, while also presenting higher impulsivity than both the BSD and BSD + NSSI groups. No differences in impulsivity between the BSD and BSD + NSSI groups were found.

**Conclusions:** The results show that BSD + NSSI and BSD + SA share a common profile characterized by difficulties in emotion regulation and low reward dependence, but differ in impulsivity and cooperativeness. This suggests that self-injury, in patients without a history of suicide attempts (i.e., BSD + NSSI), may have a regulatory role rather than being due to impulsivity.

## Introduction

Both non-suicidal self-injuries (NSSI) and suicide attempts (SA) lie along a spectrum of self-destructive actions (Nock, 2014). Although NSSI and SA share similarities in their risk factors (Andover et al., 2012), and a history of NSSI increases risk for suicide (Claes et al., 2010b), these two behaviors are distinct from each other in a number of ways. Namely, whereas NSSI represents a maladaptive coping mechanism to regulate emotions, a suicide attempt reflects a desire to end one's life (Butler et al., 2013). Hence, the intention to die is the most basic distinction between these behaviors (Andover et al., 2012). Additionally, patients who have a history of NSSI have more positive attitudes toward life than those who have had at least one SA (Muehlenkamp and Gutierrez, 2004); therefore, NSSI can be seen as being less severe than attempted suicide as the damage is not usually life threatening (In-Albon et al., 2013).

NSSI refers to any socially unacceptable behavior involving the deliberate and direct destruction of one's body tissue without suicidal intent (Muehlenkamp, 2005; Claes and Vandereycken, 2007b). According to Svirko and Hawton (2007), the prevalence of NSSI in eating disorders (ED) varies between 13.6 and 68.1% and tends to be higher in patients with anorexia nervosa binging/purging subtype (AN-BP) or with bulimia nervosa (BN) than in patients with anorexia nervosa restrictive subtype (AN-R). Similarly, SA are up to ten times more prevalent in ED compared to healthy populations, with prevalence being the highest in purging-type disorders (Pisetsky et al., 2013).

ED patients that engage in NSSI are known to have significantly higher levels of general psychopathology (Claes et al., 2003; Claes and Vandereycken, 2007a), impulsivity (Favaro and Santonastaso, 1998; Glenn and Klonsky, 2010; Claes and Muehlenkamp, 2013), depressive symptoms (Svirko and Hawton, 2007), and emotion dysregulation (Claes et al., 2010a; Riley et al., 2016) than patients without NSSI. Although both NSSI and SA are considered to lie on a continuum of self-harm behavior, individuals with NSSI report higher self-esteem, more reasons for living, and fewer depressive symptoms than those with SA (Muehlenkamp and Gutierrez, 2007; Brausch and Gutierrez, 2010).

Concerning personality traits, self-injurious ED patients report more dysfunctional personality traits, namely higher levels of harm avoidance and lower levels of self-directedness than ED patients without NSSI (Claes et al., 2012; Islam et al., 2015). In this same vein, prior research has shown that ED patients with NSSI score significantly higher on self-reported dimensions of impulsivity (Claes et al., 2013, 2014b). In addition, self-reported impulsivity has been shown to positively correlate with the frequency and severity of NSSI and with the absence of premeditation before engaging in NSSI (Janis and Nock, 2009). Similarly, ED patients with a history of SA have also been shown to present greater levels of impulsivity and to have more impulse control problems (Stein et al., 2004; Dougherty et al., 2009; Forcano et al., 2009), depression (Dougherty et al., 2009), and overall psychopathology (Favaro and Santonastaso, 1997) than ED patients without SA. Nevertheless, some studies in non-clinical samples link SA to negative affect rather than to impulsivity (Yen et al., 2009). Though personality traits have been related to SA in non-clinical samples (Brezo et al., 2008), one study examining personality traits in women with a lifetime history of ED found that SA were not significantly related to personality characteristics. However, it is important to note that the evaluated personality traits were not those most commonly associated with SA (Pisetsky et al., 2013).

The current literature suggests that both disturbed eating behavior and NSSI serve as an emotion regulation strategy (Sim and Zeman, 2006; Klonsky, 2007; Selby et al., 2008; Muehlenkamp et al., 2009, 2012b; Anestis et al., 2010; Claes et al., 2010a). NSSI is regarded as a response for managing or inhibiting aversive emotions, thus representing a dysfunctional emotion regulation strategy (In-Albon et al., 2013). NSSI in ED patients is characterized by the presence of high-activation negative affective states before engaging in NSSI behaviors (Paul et al., 2002; Claes and Vandereycken, 2007a; Klonsky, 2007; Claes et al., 2010a). For this reason, many individuals may carry out NSSI in order to find relief from negative emotions or to distract themselves from thoughts that generate negative emotions (Sim and Zeman, 2006; Klonsky, 2009; Muehlenkamp et al., 2009; Claes et al., 2010a; Riley et al., 2016).

Notwithstanding the foregoing, studies investigating the relationship between emotion regulation and SA in ED patients remain scarce and there is a lack of knowledge on the implications of personality traits and impulsivity in ED patients with SA. The rationale for splitting the self-harm behaviors into two separated groups was two-fold. First, prior research has focused mainly on investigating the differences between ED patients with and without self-harm behaviors (see Stein et al., 2004, where the mixed group is called “parasuicidal patients”), suggesting an increased tendency toward impulsivity in parasuicidal ED patients. However, these interesting results do not allow attributing differences to a specific behavior (namely NSSI or SA). In addition, some studies have identified impulsivity and personality traits that relate to SA, but were conducted in a non-clinical population (Brezo et al., 2008; Yen et al., 2009). The intention of the study at hand was to test whether these findings also apply to ED and to examine the shared influence of personality traits, impulsivity and emotion regulation on NSSI and SA. Second, identifying the differences between these two groups could be useful for helping clinicians to select the best treatment options for affected patients, especially those treatments targeting the capacity to cope with emotions in order to prevent suicide attempts.

Taking into account these aforementioned factors, the main aim of this study was to examine differences in personality traits, impulsivity, and emotion regulation between patients with ED clustered into three groups: (a) bulimic-spectrum disorders (BSD) presenting at least one lifetime SA (BSD + SA), (b) BSD patients with NSSI without history of suicide attempts (BSD + NSSI), and (c) BSD patients without a history of SA nor NSSI (BSD). Expanding upon prior research, we hypothesized that patients with a history of SA or NSSI would report greater difficulties in emotion regulation and higher scores on impulsivity scales than BSD patients without these behaviors. We also hypothesized that these two groups would show significant differences in personality traits, namely higher harm avoidance and lower self-directedness than BSD patients. We furthermore, expected that both groups with self-harm behaviors (i.e., BSD + NSSI and BSD + SA) would report differences in disorder severity, depression, impulsivity, and emotion regulation strategies, more specifically, we expected the SA group to have a more severe and more pronounced dysfunctional profile.

## Methods

### Participants and procedure

The sample comprised 122 adult female BSD patients, all of whom were characterized by the presence of binging and/or purging episodes. 9.8% (*n* = 12) were diagnosed with AN-BP, 50.8% (*n* = 62) with BN, 15.6% (*n* = 19) with binge eating disorder (BED) and 23.8% (*n* = 29) with Other Specified Feeding or Eating Disorders (OSFED). Patients were recruited via consecutive referrals to the Eating Disorders Unit at Bellvitge University Hospital (Barcelona, Spain) between December 2013 and January 2015. Diagnoses were originally made using the *Diagnostic and Statistical Manual for Mental Disorders* (*DSM-IV-TR*; APA, 2000) by means of a semi-structured interview (*SCID-I*; First et al., 2002) conducted by trained psychologists and psychiatrists. The SCID-I is a validated and commonly used interview with an inter-rater reliability of kappa 0.61 (Lobbestael et al., 2011). More specifically, inter-rater reliability for AN and BN are kappa 0.72 and kappa 0.84, respectively. Diagnoses were recodified *post-hoc* using *DSM-5* criteria (APA, 2013). For example, with DSM-5 criteria, an originally diagnosed case of eating disorders not otherwise specified (EDNOS) would be reclassified as BN if the frequency of binging or purging was at least once a week for 3 months. According to DSM-5, purging and non-purging BN were reclassified as a unique category named BN. Finally, EDNOS patients that met criteria for AN and presented a body mass index (BMI) between 17.0 and 18.5 kg/m2 were reclassified as AN. The researchers and the clinical psychologists involved in this study reviewed all diagnosed cases and diagnoses were agreed upon in a consensus meeting.

From an initial sample of 155 ED patients, the following groups were excluded: (a) AN-R patients (*n* = 24). This was carried out in order to homogenize the sample, since prior research has identified different personality profiles between AN-R and AN-BP patients, as well as differences in the prevalence of NSSI and SA between these groups (Ahrén-Moonga et al., 2008); and (b) males (*n* = 9) as the number of men with ED in our sample was too small for meaningful comparison.

In accordance with our study aims, patients were clustered *post-hoc* into three mutually exclusive groups: BSD patients without current or lifetime NSSI or SA (BSD; *n* = 57), BSD patients who had engaged in NSSI at least once in their lifetime but never had attempted suicide (BSD + NSSI; *n* = 28), and BSD patients with at least one lifetime suicide attempt (BSD + SA; *n* = 37). This last group labeled as BSD + SA comprised 16 patients without lifetime NSSI (43.2%) and 21 patients with lifetime NSSI (56.8%).

The Ethics Committee of our Institution (the Ethics Committee of Clinical Research at the University Hospital of Bellvitge) approved the study, and all research was conducted in accordance with the latest version of the Helsinki Declaration. Signed informed consent was obtained from all participants.

### Assessment

To assess impulse-control problems (including NSSI and SA), we used a checklist of nine lifetime impulsive behaviors, such as stealing, pathological gambling, NSSI, SA, aggressions toward objects, hetero-aggression, alcohol abuse, drug abuse, and tobacco use. All the patients were asked whether each behavior was present or absent (current or lifetime) during a semi-structured clinical interview conducted by experienced psychologists and psychiatrists. The presence/absence of NSSI was investigated by a 1-item question asking “Have you ever engaged in self-injury without the intent to die (such as self-cutting, burning, hitting, scratching, and hair pulling)?” Drug or alcohol abuse, binging or vomiting behaviors were not included as NSSI. Using a single-item measure is common in NSSI research and has been shown to render consistent estimates of prevalence (Claes et al., 2012; Muehlenkamp et al., 2012a; Islam et al., 2015).

The presence/absence of suicide attempts was assessed by the question: “Have you ever attempted suicide (with lethal intentionality)? In case of an affirmative response, explain how.”

**Eating Disorders Inventory-2 (EDI-2)** (Garner, 1991). The EDI-2 is a self-report questionnaire that assesses characteristics of eating disorders through 91 items on the dimensions: drive for thinness, bulimia, body dissatisfaction, ineffectiveness, perfectionism, interpersonal distrust, interoceptive awareness, maturity fears, asceticism, impulse regulation, and social insecurity. A Spanish version of this questionnaire was validated (Garner, 1998). Internal consistency was excellent in our sample (α = 0.95).

**Symptom Check-List 90 Revised (SCL-90-R)** (Derogatis, 1990). The SCL-90-R is a self-report questionnaire that measures psychological distress and psychopathology through 90 items on the following dimensions: somatization, obsessive-compulsive, interpersonal sensitivity, depression, anxiety, hostility, phobic anxiety, paranoid ideation, and psychoticism. The global score (Global Severity Index, GSI) is widely used to measure psychopathological distress. The Spanish version of the revised questionnaire was validated (Derogatis, 2002). Internal consistency was excellent in our sample (α = 0.98).

**Temperament and Character Inventory Revised (TCI-R)** (Cloninger, 1994). The TCI-R is a 240-item self-report questionnaire that measures four temperament (namely novelty seeking, harm avoidance, reward dependence and persistence), and three character dimensions of personality (namely self-directedness, cooperativeness and self-transcendence). The Spanish version of the revised questionnaire was validated and shown to have good psychometric properties (internal consistency of α = 0.87; Gutiérrez-Zotes et al., 2004). Cronbach's alpha for the current sample was good (α = 0.75 for “novelty seeking”) to excellent (α = 0.92 for “harm avoidance”).

**UPPS Impulsive Behavior Scale (UPPS-P)** (Whiteside and Lynam, 2001; Whiteside et al., 2005). The UPPS-P is a 59-item self-report inventory that measures five distinct facets of impulsiveness: negative urgency (i.e., the tendency to act rashly in response to distress), lack of perseverance (i.e., the inability to remain focused on a task), lack of premeditation (i.e., the tendency to act without thinking of the consequences of one's actions), sensation seeking (i.e., the tendency to seek out novel and thrilling experiences), and positive urgency (i.e., the tendency to act rashly in response to positive mood). Each item is rated on a 4-point Likert scale ranging from 1 “strongly agree” to 4 “strongly disagree.” This questionnaire has been validated in a Spanish population and has good psychometric properties (Verdejo-García et al., 2010). Internal consistency for the current sample was in the good to excellent range (Cronbach's alpha 0.79 for “lack of premeditation” and 0.90 for “sensation seeking”).

**Barratt Impulsiveness Scale - 11 (BIS-11)** (Patton et al., 1995). The BIS-11 is a self-report 30-item scale that measures impulsivity. Each item is rated on a 4-point Likert scale ranging from 1 (rarely/never) to 4 (almost often). Items are divided over three subscales: cognitive impulsiveness (inattention, cognitive instability), motor impulsiveness (acting spontaneously and lack of perseverance), and non-planning impulsiveness (lack of premeditation, intolerance of cognitive complexity, and lack of self-control). Total scores can range from 30 to 120, with higher scores indicating greater impulsiveness levels. The Spanish version of the BIS-11 was validated in an adult population (Oquendo et al., 2001), with adequate internal consistency in the current sample (α = 0.72).

**Difficulties in Emotion Regulation Scale (DERS)** (Gratz and Roemer, 2004). The DERS assesses emotion dysregulation across six subscales: non-acceptance of emotional responses; difficulties in pursuing goals when having strong emotions; difficulties controlling impulsive behaviors when experiencing negative emotions; lack of emotional awareness; limited access to emotion regulation strategies; and lack of emotional clarity. Higher scores indicate more difficulties in emotion regulation. The Spanish version of the DERS was validated in an adult population and showed adequate construct and predictive validity, along with excellent internal consistency (Cronbach's alpha, α = 0.96; Wolz et al., 2015). Internal consistency for the current sample was excellent (α = 0.94) for DERS total scores.

### Statistical data analysis

Statistical analysis was carried out using SPSS 20 for Windows. Analysis of variance (ANOVA) procedures compared the means of clinical, psychopathological, and personality variables between groups (BSD, BSD + NSSI, and BSD + SA), defining *post-hoc* multiple contrasts through Bonferroni estimations to measure pairwise comparisons. To compare categorical variables, we used Chi-square tests. Significance level was set at *p* < 0.05. In cases of multiple comparisons, the Bonferroni correction method has largely been criticized for being too conservative. From a practical-clinical perspective, effect sizes are the relevant objective of the analyses (*p*-values are strongly dependent on sample sizes) and thus, all the effect sizes for the relationships analyzed in this study have been estimated to correct for the use of multiple group comparisons. For pairwise comparisons, effect sizes were measured through Cohen's *d* coefficient (|*d*| < 0.5 was considered poor, |*d*| > 0.5 was considered moderate and |*d*| > 0.8 was considered large). In this study, to avoid the risk of false positives due to the presence of multiple comparison tests, only mean differences which surpassed the predetermined statistical significance threshold, and which obtained effect sizes in the moderate to large range were considered as relevant.

## Results

### Description of sample

The mean age of the whole sample was 28.57 years old (*SD* = 9.45), with no significant age-related differences being found between groups. Socio-demographic characteristics, as well as information on the presence of impulsive behaviors are described in Table 1. No statistical differences between groups emerged concerning socio-demographic characteristics such as education level, civil status and employment status. The groups showed no differences in impulse-control problems, with the exception of alcohol abuse. Patients in the BSD + SA group had a significantly higher prevalence of alcohol abuse than the BSD group (*p* = 0.018 and *d* = 0.52).

**Table 1:** **Sample description**.

		**Total (*n*** = **122) (%)**	**BSD (*n*** = **57) (%)**	**BSD + NSSI (*n*** = **28) (%)**	**BSD + SA (*n*** = **28) (%)**	**χ^2^**	***df***	***p***
Education level	Primary	43.4	36.8	50.0	48.6	3.11	4	0.539
	Secondary	38.5	45.6	35.7	29.7			
	University	18.0	17.5	14.3	21.6			
Civil status	Single	72.1	73.7	78.6	64.9	2.00	4	0.735
	Married-partner	20.5	19.3	17.9	24.3			
	Divorced-separated	7.4	7.0	3.6	10.8			
Employment status	Employed	72.1	75.4	82.1	59.5	4.66	2	0.097
Stealing behavior		44.3	35.1	46.4	56.8	4.34	2	0.114
Gambling behavior		4.1	3.5	0	8.1	2.76	2	0.252
Aggressions toward objects		19.7	15.8	17.9	27.0	1.87	2	0.393
Aggressions toward other people		5.7	1.8	7.1	10.8	3.54	2	0.171
Alcohol abuse		13.1	7.0	10.7	24.3	6.08	2	0.048
Drug use/abuse		16.4	14.0	10.7	24.3	2.59	2	0.274
Tobacco use		36.1	35.1	39.3	35.1	0.16	2	0.922
BSD subtype	AN-BP	9.8	5.3	14.3	13.5	12.15	6	0.059
	BN	50.8	42.1	57.1	59.5			
	BED	15.6	26.3	7.1	5.4			
	OSFED	23.8	26.3	21.4	21.6			

*BSD, bulimic-spectrum disorder; NSSI, non-suicidal self-injury; SA, suicide attempts; AN-BP, anorexia nervosa binging/purging subtype; BN, bulimia nervosa; BED, binge eating disorder; OSFED, Other Specified Feeding or Eating Disorders. Comparison between groups based on chi-square tests*.

### Eating and general psychopathology

As shown in Table 2, the three groups did not statistically differ in the frequency of binging and compensatory behaviors (such as purging and laxative use), and disorder duration. The only difference found was regarding body mass index (BMI), with the BSD group presenting higher BMI than those with SA. Regarding eating pathology as measured by the EDI-2, we found that the BSD + NSSI and BSD + SA groups showed higher BSD psychopathology than the BSD group (considering EDI-2 total scores). Differences in subscale scores can be found in Table 2.

**Table 2:** **Comparison of clinical variables**.

		**Mean (standard deviation)**								**Pairwise comparisons**					
		**BSD (***n* = **57)**		**BSD** + **NSSI** (*n* = **28)**		**BSD** + **SA** (*n* = **37)**		**Factor group**		**BSD vs**. **BSD** + **NSSI**		**BSD vs**. **BSD** + **SA**		**NSSI vs**. **SA**	
		**Mean**	***SD***	**Mean**	***SD***	**Mean**	***SD***	***F_df_*** = **119**	***p***	**MD^a^**	***|d|*^b^**	**MD^a^**	***|d|*^b^**	**MD^a^**	***|d|*^b^**
Age (years-old)		29.30	10.04	26.29	7.64	29.19	9.71	1.07	0.347	3.01	0.34	0.11	0.01	−2.90	0.33
Age of onset (years-old)		21.16	8.36	18.11	6.34	19.16	9.32	1.46	0.237	3.05	0.41	2.00	0.23	−1.06	0.13
Duration of BSD (years)		8.06	6.99	8.36	6.72	10.04	8.31	0.86	0.427	−0.30	0.04	−1.99	0.26	−1.68	0.22
Number of previous treatments		0.53	0.78	0.69	1.19	0.76	0.86	0.79	0.455	−0.17	0.16	−0.23	0.28	−0.06	0.06
Binges per week		4.23	3.97	2.86	2.88	4.73	6.11	1.43	0.244	1.37	0.40	−0.50	0.10	−1.87	0.39
Vomits per week		3.48	5.21	3.82	3.99	5.36	10.24	0.86	0.427	−0.34	0.07	−1.88	0.23	−1.54	0.20
Laxatives per week		2.75	7.01	2.52	6.97	2.22	4.83	0.08	0.924	0.24	0.03	0.54	0.09	0.30	0.05
BMI (kg/m2)		28.23	10.09	24.65	6.89	23.84	6.55	3.51	0.033	3.58	0.41	**4.39**	**0.52**	0.81	0.12
EDI: Drive for thinness	α = 0.77	14.96	5.24	17.04	3.43	14.70	5.21	2.17	0.118	−**2.07**	**0.50**	0.26	0.05	**2.33**	**0.53**
EDI: Body dissatisfaction	α = 0.87	18.21	7.89	19.00	6.69	18.59	7.63	0.11	0.899	−0.79	0.11	−0.38	0.05	0.41	0.06
EDI: Interoceptive Awareness	α = 0.78	10.42	5.82	13.86	6.97	14.08	6.90	4.67	0.011	−**3.44**	**0.53**	−**3.66**	**0.57**	−0.22	0.03
EDI: Bulimia	α = 0.77	8.77	5.34	8.93	5.08	7.57	5.09	0.76	0.471	−0.16	0.03	1.20	0.23	1.36	0.27
EDI: Interpersonal Distrust	α = 0.77	4.00	3.26	6.89	4.75	8.22	4.94	12.33	<0.001	−**2.89**	**0.71**	−**4.22**	**1.01**	−1.32	0.27
EDI: Ineffectiveness	α = 0.87	8.58	5.73	13.75	7.03	17.05	7.94	18.41	<0.001	−**5.17**	**0.81**	−**8.48**	**1.22**	−3.30	0.44
EDI: Maturity fears	α = 0.80	8.21	5.25	8.75	6.95	10.24	6.39	1.30	0.277	−0.54	0.09	−2.03	0.35	−1.49	0.22
EDI: Perfectionism	α = 0.73	4.93	4.50	6.89	4.39	5.92	4.19	1.96	0.145	−1.96	0.44	−0.99	0.23	0.97	0.23
EDI: Impulse regulation	α = 0.82	4.81	5.75	7.50	5.85	10.14	7.28	8.18	<0.001	−2.69	0.46	−**5.33**	**0.81**	−2.64	0.40
EDI: Ascetism	α = 0.65	6.14	3.74	8.32	3.13	9.35	5.02	7.58	0.001	−**2.18**	**0.63**	−**3.21**	**0.72**	−1.03	0.25
EDI: Social insecurity	α = 0.83	5.70	4.35	10.46	5.77	10.86	5.75	14.34	<0.001	−**4.76**	**0.93**	−**5.16**	**1.01**	−0.40	0.07
EDI: Total score	α = 0.95	94.74	36.12	121.39	36.50	126.73	46.17	8.72	<0.001	−**26.7**	**0.73**	−**32.0**	**0.77**	−5.34	0.13

*BSD, bulimic-spectrum disorder; NSSI, non-suicidal self-injury; SA, suicide attempts; BMI, body mass index; |d|, Cohen's-d for effect size*.

a *Bold, significant pairwise comparison (0.05 level)*.

b *Bold, moderate (|d| > 0.5) to large (|d| > 0.8) effect size*.

*α, Chronbach's alpha in the sample. Results obtained via ANOVA procedure*.

Regarding general psychopathology as measured by the SCL-90R, our results found that the BSD + SA group exhibited significantly greater psychopathology than the BSD group. Likewise, the BSD + NSSI group also presented higher scores in obsessive-compulsive symptoms, depression and total GSI scores than the BSD group without NSSI. There were no statistically significant differences between BSD + NSSI and BSD + SA regarding general psychopathology (see Table 3).

**Table 3:** **Comparison of psychopathological variables**.

		**Mean (standard deviation)**								**Pairwise comparisons**					
		**BSD (*****n*** = **57)**		**BSD** + **NSSI (*****n*** = **28)**		**BSD** + **SA (*****n*** = **37)**		**Factor group**		**BSD vs**. **BSD** + **NSSI**		**BSD vs**. **BSD** + **SA**		**NSSI vs**. **SA**	
	**α**	**Mean**	***SD***	**Mean**	***SD***	**Mean**	***SD***	***F_df_*** = **119**	***p***	**MD^a^**	***|d|*^b^**	**MD^a^**	***|d|*^b^**	**MD^a^**	***|d|*^b^**
SCL-90: Somatization	0.89	1.71	0.84	2.10	0.85	2.22	1.01	4.02	0.020	−0.39	0.46	−**0.50**	**0.54**	−0.12	0.13
SCL-90: Obsessive/compulsive	0.83	1.69	0.73	2.06	0.63	2.24	0.90	6.21	0.003	−**0.37**	**0.54**	−**0.55**	**0.67**	−0.18	0.23
SCL-90: Interpersonal sensitivity	0.87	1.94	0.90	2.19	0.90	2.51	0.91	4.47	0.013	−0.25	0.28	−**0.57**	**0.63**	−0.31	0.35
SCL-90: Depressive	0.90	1.94	0.83	2.39	0.81	2.75	0.90	10.69	<0.001	−**0.45**	**0.56**	−**0.81**	**0.94**	−0.36	0.42
SCL-90: Anxiety	0.85	1.48	0.70	1.80	0.70	2.08	1.00	6.44	0.002	−0.33	0.46	−**0.60**	**0.70**	−0.28	0.32
SCL-90: Hostility	0.86	1.22	0.87	1.38	0.78	1.82	1.06	4.95	0.009	−0.16	0.19	−**0.60**	**0.62**	−0.44	0.48
SCL-90: Phobic anxiety	0.81	0.84	0.76	1.14	0.85	1.46	1.10	5.39	0.006	−0.30	0.37	−**0.62**	**0.65**	−0.32	0.32
SCL-90: Paranoia	0.75	1.45	0.76	1.56	0.60	1.66	0.92	0.78	0.462	−0.11	0.15	−0.20	0.24	−0.10	0.13
SCL-90: Psychotic	0.82	1.23	0.69	1.48	0.67	1.68	0.87	4.08	0.019	−0.25	0.36	−**0.45**	**0.57**	−0.20	0.25
SCL-90: GSI score	0.98	1.59	0.64	1.91	0.59	2.16	0.79	7.87	0.001	−**0.31**	**0.51**	−**0.56**	**0.78**	−0.25	0.36
SCL-90: PST score	0.98	63.61	15.56	70.29	14.64	71.16	16.46	3.22	0.044	−6.67	0.44	−**7.55**	**0.50**	−0.88	0.06
SCL-90: PSDI score	0.98	2.19	0.56	2.41	0.45	2.64	0.61	7.50	0.001	−0.22	0.43	−**0.45**	**0.77**	−0.23	0.44

*BSD, bulimic-spectrum disorder; NSSI, non-suicidal self-injury; SA, suicide attempts; SCL-90, Symptom Checklist 90; GSL, Global Severity Index; PST, Positive Symptom Total. Positive Symptom Distress Index. |d|, Cohen's-d for effect size*.

a *Bold, significant pairwise comparison (0.05 level)*.

b *Bold, moderate (|d| > 0.5) to large (|d| > 0.8) effect size*.

*α, Chronbach's alpha in the sample. Results obtained via ANOVA procedure*.

### Personality traits, impulsivity, and difficulties in emotion regulation

Regarding personality traits, patients in the BSD with SA or NSSI groups obtained significantly lower reward dependence scores (i.e., low tendency to respond markedly to signals of reward, particularly social support or approval) than only-BSD patients. Moreover, BSD + SA patients had significantly lower scores in persistence (this personality trait refers to perseverance in spite of fatigue or frustration) than BSD patients. BSD + NSSI and BSD + SA groups differed in cooperativeness (i.e., relating to individual differences in how much people identify with and accept others), with the BSD + SA group presenting lower scores in this trait.

With respect to impulsivity, participants in the BSD + SA group showed higher scores on the UPPS-P lack of perseverance subscale than the BSD group. BSD + NSSI and BSD + SA significantly differed in the lack of premeditation subscale, with BSD + SA having the highest score. In addition, the BSD + SA group demonstrated higher total impulsiveness, measured by BIS-11, in comparison with both BSD and BSD + NSSI groups. BSD + SA also showed higher scores on cognitive impulsiveness than BSD patients, and higher non-planning impulsiveness than the BSD + NSSI group. Effect sizes for these statistical differences were in the moderate range (see Table 4).

**Table 4:** **Comparison of personality traits, impulsivity and emotion regulation measures**.

		**Mean (standard deviation)**								**Pairwise comparisons**					
		**BSD (*****n*** = **57)**		**BSD** + **NSSI (*****n*** = **28)**		**BSD** + **SA (*****n*** = **37)**		**Factor group**		**BSD vs**. **BSD** + **NSSI**		**BSD vs**. **BSD** + **SA**		**NSSI vs**. **SA**	
	**α**	**Mean**	**SD**	**Mean**	**SD**	**Mean**	**SD**	***F_df_*** = **119**	***p***	**MD^a^**	***|d|*^b^**	**MD^a^**	***|d|*^b^**	**MD^a^**	***|d|*^b^**
TCI-R: Novelty seeking	0.75	105.79	15.53	101.68	11.25	101.68	16.21	1.16	0.317	4.11	0.30	4.11	0.26	0.00	0.00
TCI-R: Harm avoidance	0.92	114.44	20.25	118.93	21.71	123.32	22.61	1.97	0.144	−4.49	0.21	−**8.89**	0.41	−4.40	0.20
TCI-R: Reward dependence	0.80	107.12	15.12	97.46	13.61	96.54	15.01	7.27	0.001	**9.66**	**0.67**	**10.58**	**0.70**	0.92	0.06
TCI-R: Persistence	0.87	109.37	21.25	104.32	19.10	97.57	18.30	3.94	0.022	5.05	0.25	**11.80**	**0.60**	6.75	0.36
TCI-R: Self-directedness	0.85	117.72	20.55	116.71	17.30	109.00	20.56	2.32	0.102	1.01	0.05	**8.72**	**0.52**	7.71	0.41
TCI-R: Cooperativeness	0.84	134.84	14.33	138.46	17.02	128.24	19.09	3.32	0.039	−3.62	0.23	6.60	0.39	**10.22**	**0.57**
TCI-R: Self-transcendence	0.83	66.25	13.90	65.21	11.22	62.08	16.93	0.96	0.385	1.03	0.08	4.16	0.27	3.13	0.22
UPPS: Negative urgency	0.86	34.18	5.99	34.64	6.09	34.28	6.14	0.06	0.946	−0.46	0.08	−0.10	0.02	0.37	0.06
UPPS: Lack premeditation	0.79	23.63	5.93	22.46	5.33	25.92	6.51	2.88	0.060	1.16	0.21	−2.29	0.37	−**3.45**	**0.58**
UPPS: Lack Perseverance	0.88	22.48	4.99	24.75	5.52	25.28	4.55	4.03	0.020	−2.27	0.43	−**2.80**	**0.59**	−0.53	0.10
UPPS: Sensation seeking	0.90	27.00	8.67	25.96	9.91	25.44	7.99	0.37	0.692	1.04	0.11	1.56	0.19	0.52	0.06
UPPS: Positive urgency	0.80	30.11	8.80	29.32	7.78	31.31	10.07	0.40	0.668	0.79	0.09	−1.20	0.13	−1.98	0.22
DERS: Non-acceptance	0.89	17.58	6.16	21.43	6.20	20.70	6.84	4.51	0.013	−**3.85**	**0.62**	−**3.12**	**0.50**	0.73	0.11
DERS: Goals	0.87	15.82	4.23	18.86	4.87	18.14	5.20	4.93	0.009	−**3.03**	**0.66**	−**2.31**	**0.50**	0.72	0.14
DERS: Impulse	0.88	15.54	6.31	17.43	6.72	19.22	5.56	4.00	0.021	−1.88	0.29	−**3.67**	**0.62**	−1.79	0.29
DERS: Awareness	0.77	17.35	5.32	17.54	3.91	19.43	4.96	2.18	0.117	−0.18	0.04	−**2.08**	**0.51**	−1.90	0.42
DERS: Strategies	0.88	23.28	6.85	27.61	8.97	27.46	7.88	4.62	0.012	−**4.33**	**0.54**	−**4.18**	**0.57**	0.15	0.02
DERS: Clarity	0.85	14.68	4.66	15.75	4.92	17.41	4.34	3.88	0.023	−1.07	0.22	−**2.72**	**0.60**	−1.66	0.36
DERS: Total score	0.94	104.26	22.35	118.61	28.17	122.35	23.96	7.19	0.001	−**14.3**	**0.56**	−**18.1**	**0.78**	−3.74	0.14
BIS: Cognitive	0.70	15.63	4.47	16.54	3.37	18.19	4.61	3.99	0.021	−0.90	0.23	−**2.56**	**0.56**	−1.65	0.41
BIS: Motor	0.70	18.84	7.36	17.57	6.75	20.86	7.78	1.70	0.187	1.27	0.18	−2.02	0.27	−3.29	0.45
BIS: No plan	0.73	19.28	6.42	18.29	5.57	21.51	6.96	2.28	0.107	0.99	0.17	−2.23	0.33	−**3.23**	**0.51**
BIS: Total	0.72	53.75	13.60	52.39	11.13	60.57	14.22	3.95	0.022	1.36	0.11	−**6.81**	**0.50**	−**8.17**	**0.64**

*BSD, bulimic-spectrum disorder; NSSI, non-suicidal self-injury; SA, suicide attempts; TCI-R, Temperament and Character Inventory revised; UPPS-P, Impulsive Behavior Scale; BIS, Barratt Impulsiveness Scale – 11; DERS, Difficulties in Emotion Regulation Scale; DERS Non-acceptance, Non-acceptance of emotional responses; DERS Goals, Difficulties engaging in goal directed behavior; DERS Impulse, Impulse control difficulties; DERS Awareness, Lack of emotional awareness; DERS Strategies, limited access to emotion regulation strategies; DERS Clarity, lack of emotional clarity |d| Cohen's-d for effect size*.

a *Bold, significant pairwise comparison (0.05 level)*.

b *Bold, moderate (|d| > 0.5) to large (|d| > 0.8) effect size*.

*α, Chronbach's alpha in the sample. Results obtained via ANOVA procedure*.

Regarding emotion dysregulation, both BSD + NSSI and BSD + SA groups had higher scores than the BSD group on the following DERS subscales: non-acceptance of emotional responses, difficulties engaging in goal-directed behavior, limited access to emotion regulation strategies, and total DERS scores. Moreover, the BSD + SA group scored higher than BSD in almost all of the DERS subscales, except for the lack of emotional awareness subscale. There were no significant differences in DERS scores between the BSD + NSSI and the BSD + SA groups. Effect sizes were in the moderate range (shown in Table 4).

## Discussion

The main aim of this study was to examine differences in personality traits, impulsivity, and emotion regulation between patients with BSD only, BSD with SA and BSD with NSSI. Although no differences were found among the three groups in terms of the frequency of bulimic symptoms and disorder duration, both groups presenting self-harm behaviors (i.e., BSD + NSSI and BSD + SA) reported significantly higher levels of disordered eating and psychopathology than the BSD group. These results are consistent with existing research suggesting that BSD patients with NSSI or SA present greater levels of psychopathology, and therefore, more disorder severity (Claes et al., 2003; Claes and Vandereycken, 2007a; Svirko and Hawton, 2007; Islam et al., 2015). Virtually no differences were found between BSD + NSSI and BSD + SA groups regarding eating psychopathology. Surprisingly, body dissatisfaction didn't differ between groups, which is in contrast to prior research (Muehlenkamp et al., 2012b). High scores in ineffectiveness and interoceptive awareness in BSD patients with NSSI or SA were also found. These characteristics have previously been linked to problems recognizing and facing emotional states (Sim and Zeman, 2006), or difficulties verbally describing emotions, and are common features in alexithymia (Garner et al., 1983; Taylor et al., 1996).

Regarding personality traits, our results found that both BSD + NSSI and BSD + SA groups had similarly low scores in reward dependence in comparison with the BSD group. These findings are in line with a recently published study suggesting that patients with self-harm behaviors present a tendency toward introversion (Islam et al., 2015). However, in our study the BSD + NSSI group showed significantly higher scores on cooperativeness than the BSD + SA group; suggesting that, while the former have a desire to please others, the latter have an inclination toward social disinterest and selfishness (Cloninger et al., 1993). It further suggests that BSD + NSSI feel more connected to their social environment than BSD + SA, wherefore their self-injurious behavior may function as a cry for help, while BSD + SA may not believe that significant others are able to help them. Nevertheless, the present study was not able to determine whether these traits compel BSD patients to engage in NSSI or SA or if engaging in these behaviors isolates them from others, because of feelings of shame or guilt, as prior research has reported (Mckenzie and Gross, 2014). Unlike previous studies (Claes et al., 2012), the present study found no differences between groups in harm-avoidance and self-directedness. However, a large-scale population based study on the correlates of suicide in ED did not find an association between lower self-directedness in individuals with ED and suicide attempts (Pisetsky et al., 2013). One possible explanation could be that this trait is characteristic of the ED in itself (Aguera et al., 2012) and therefore, low self-directedness may be better explained by the presence of an eating disorder and not by self-harm behaviors. Surprisingly, only BSD + SA patients reported more impulsivity than the other two groups (namely greater cognitive impulsiveness and lack of perseverance). These findings suggest that greater impulsivity may differentiate BSD patients with SA from those with NSSI or only BSD. These findings are consistent with prior studies indicating that impulsivity does not differ between ED patients who engage in NSSI from those who do not (Newton et al., 1993; Claes et al., 2014a; Islam et al., 2015). However, our results are not consistent with those of other authors indicating that self-injurers were best described by high levels in UPPS-P impulsivity (Janis and Nock, 2009; Glenn and Klonsky, 2010; Claes and Muehlenkamp, 2013). These discrepancies might be because these traits are characteristic of BSD and since our sample uniquely consisted of these patients, they are homogeneous in this facet.

In line with previous studies (Muehlenkamp et al., 2012b; Arbuthnott et al., 2015), results from the current study confirm the hypothesis that both BSD + NSSI and BSD + SA patients present more difficulties in emotion regulation than patients with only BSD. Specifically, it was found that both groups were more likely to report difficulties accepting distressing emotions, concentrating and accomplishing tasks when experiencing negative emotions, as well as believing that there is little that can be done to regulate emotions effectively once they are upset (Gratz and Roemer, 2004). These findings suggest that NSSI is better explained by impairments in emotion regulation rather than by impulsivity (Favaro and Santonastaso, 1998; Claes et al., 2014a; Islam et al., 2015; Turner et al., 2015). This is particularly relevant since it supports the hypothesis that self-harm behaviors act as regulators of negative affective states (Paul et al., 2002; Claes and Vandereycken, 2007a; Klonsky, 2007; Claes et al., 2010a; Riley et al., 2016).

## Limitations and strengths

The results of this study should be evaluated within the context of several limitations. First, the data are self-reported and cross-sectional, thus limiting causal conclusions. Longitudinal studies with treatment data are needed to fully substantiate the present conclusions. Second, NSSI was only measured as a dichotomous variable indicating the absence or presence of lifetime NSSI. In future research, NSSI may be assessed more extensively including the frequency of NSSI and/or different types of NSSI (Claes et al., 2010a). Third, all participants in this study were women with BSD mainly due to the small number of male ED patients in our sample. Thus, these findings cannot be generalized to males or other psychiatric populations.

In spite of these limitations, this study has several strengths. To our knowledge, this is the first study to compare emotion regulation, impulsiveness and personality traits in BSD + NSSI patients and BSD + SA patients. These findings could be useful in identifying different profiles between these groups and providing a better understanding of the relationship between these variables in BSD patients. Methodologically, the study results are strengthened by the use of different scales to assess impulsivity, allowing for the identification of what dimensions of impulsiveness are most related to NSSI and SA. Furthermore, all patients were consecutive referrals and our sample did not include patients with subclinical eating disorders.

## Clinical implications

The current findings have numerous implications for clinical practice. First, the present results point to the potential utility of interventions utilizing emotion-based treatments like dialectical behavior therapy (DBT) and acceptance-based emotion regulation group therapy (Gratz, 2007) to treat ED patients presenting both NSSI and SA. Moreover, cognitive behavioral therapy (CBT) could be used to teach these patients strategies to express their thoughts and feelings (Aguera et al., 2012). Novel approaches combining CBT with new technology [Cognitive Remediation and Emotion Skills Training (CREST)] might be a beneficial in treating ED patients with NSSI or SA, since these combined treatments have proved to be effective at improving emotional regulation skills (Fagundo et al., 2014; Tchanturia et al., 2015). In the light of these results, BSD + SA patients would most benefit from treatments focused on managing impulsivity, for example, by means of neuro-feedback and/or biofeedback techniques that increase physiological control, or cognitive training strategies to reduce arousal and to improve decision making and planning (e.g., the Playmancer serious video game; Fernandez-Aranda et al., 2012). NSSI patients on the other hand might benefit more from emotion regulation skills training than from treatments focused on impulsivity and inhibitory control. Training NSSI patients to recognize and manage emotions could help to reduce self-harm behavior. Longitudinal studies are needed to compare the effectiveness of each approach.

Another important implication of this study concerns the low levels of social cooperativeness observed in BSD + SA patients. These results highlight that these patients are in special need of interventions focusing on improving of social bonds and increasing feelings of connectedness with others.

## Conclusions

The results of the present study highlight the complex relationship between self-harm (NSSI or SA), emotion regulation, impulsivity and personality traits in BSD patients. This study provides data suggesting that BSD patients with either NSSI or SA present more difficulties to openly express their feelings and to adequately regulate their emotions than BSD patients without self-harm behaviors. BSD + SA patients may have a greater degree of social insecurity and less cooperativeness compared to those with NSSI. In sum, our findings indicate that NSSI may be better understood as a maladaptive form of emotion regulation than as an impulsive behavior.

## Author contributions

AG, IW, AF, SJ, ZA, and FF participated in the design of the study. RG was responsible for statistical analysis and for writing statistical sections of the manuscript. All authors (AG, IW, AF, TS, RG, SJ, ZA, FF) contributed to critically revising the work, approved the final version of the article to be published and agreed to be accountable for all aspects of the work in ensuring that questions related to the accuracy or integrity of any part of the work are appropriately investigated and resolved.

## Funding

The author(s) disclosed receipt of the following financial support for the research, authorship, and/or publication of this article: Preparation of this manuscript was supported by both FIS PI14/00290 and PSI2011-28349 from Instituto de Salud Carlos III (ISCIII) and Ministerio de de Economía y Competitividad, respectively. This study was also co-funded by Fondos Europeos de Desarrollo Regional (FEDER) funds - a way to build Europe. CIBERobn is an initiative of ISCIII. IW was supported by a pre-doctoral grant of Agència de Gestió d'Ajuts Universitaris i de Recerca (AGAUR) (2016FI_B2 00001).

### Conflict of interest statement

The authors declare that the research was conducted in the absence of any commercial or financial relationships that could be construed as a potential conflict of interest.
